# Doing Health Policy Analysis: The Enduring Relevance of Simple Models: Comment on "Modelling the Health Policy Process: One Size Fits All or Horses for Courses"

**DOI:** 10.34172/ijhpm.2023.8223

**Published:** 2023-12-04

**Authors:** Lucy Gilson, Gill Walt

**Affiliations:** ^1^Health Policy and Systems Division, School of Public Health, University of Cape Town, Cape Town, South Africa; ^2^Department of Global Health and Development, London School of Hygiene and Tropical Medicine, London, UK

**Keywords:** Health Policy Processes, Health Policy, Public Policy, Research

## Abstract

The analysis of health policy processes in low- and middle-income countries (LMICs) emerged as a research area in the early 1990s. In their recent editorial Powell and Mannion argue that such research can be deepened by applying public policy theory. In response, we raise three questions to consider: are public policy models fit for purpose in today’s world in LMICs (and what other theory can be used)? Is using theory the most important factor in deepening such research? Why do we, as researchers, do this work? Ultimately, we argue that the value of simple models, such as those already used in health policy analysis, lies in their enduring relevance and widespread use. They are supporting the development of the shared understandings that can, in turn, provide the basis for collective action addressing inequities in health and well-being.

 A recent editorial^1^ and responding commentaries^2-5^ have considered the conceptual models and theories used in analysis of health policy processes. These pieces have raised interesting points about the role of theory in such analysis, the bodies of theory used and how to deepen future research through the use of different theoretical lenses. Reflecting on the existing research, particularly in low- and middle-income countries (LMICs), the editorial’s authors^1^ specifically argue that this health policy analysis is “semi-detached” from wider policy process research, and could be strengthened by applying the “conceptually stronger” public policy models. Using these models would, they suggest, generate better understanding and explanations of health policy processes. The wide application of the Health Policy Analysis Triangle (HPAT) model^6^ is specifically cited as linked to the distance between health and public policy literature.

 In engaging with these discussions, we ask three sets of questions – about theory use, the deepening of health policy process research and its substantive relevance.

##  First, is the existing body of public policy theory fit-for-purpose in examining health policy processes in today’s world, in LMICs? And, if not, what other theory can be used?

 At least some of the public policy models recommended by Powell and Mannion^1^ were developed in decades past, and so it is appropriate to consider whether they adequately address the complex forces currently influencing health policy processes in LMICs and elsewhere. These include “complex cross-border, inter-organizational and network relationships, with policies influenced by global decisions as well as by domestic actions” (p. 309).^7^ Do public policy models assist, for example, in examining the commercial determinants of health that have become so influential in relation to non-communicable diseases?^8^ Do they allow for the massive changes in communication brought about by the internet and social media, that impacted on COVID-19 vaccination debates and rates?^9^ Similar questions could, of course, be asked about the relevance of these models to other areas of policy,^3^ further emphasising the need for theory to address contemporary phenomena.

 In addition, as Parkhurst^2^ notes, public policy models were largely developed in the US and Europe and do not engage with the particular contexts of LMICs. *Contemporary *political and political economy theory developed *in* LMICs is, instead, likely to offer greater value in understanding policy processes, including those in the health sector, in these contexts.

 Context-relevant theory is particularly important in studying health policy implementation, a source of many policy challenges and yet particularly under-researched in LMICs.^1^ Such research considers how implementors’ collective action is sustained or undermined over time, recognising the interdependence of implementation determinants. For example, it includes consideration of the ways in which contextual features (eg, front-line provider values and beliefs, legal regulations, governance norms) influence policies themselves, as well as implementation processes.^10^ For such research, context-specific governance insights drawn from anthropology, sociology, organizational theory, and public administration are particularly valuable.^11^

 Ultimately, then, whilst we agree that paying more attention to theory^7^ is important in deepening understanding of the chains of causality within health policy processes, we do not see public policy theory as the only, or even most, relevant theoretical base. Other commentaries^2-5^ offer a range of potentially relevant theories. We argue that the theory applied must assisting in understanding the contemporary local, national and global political and governance contexts in which policy processes unfold in LMIC settings (such as the influence of global health actors). In addition, it must support understanding of the particularities of health policy (such as the influence of clinicians and their professional associations). Such theory may comprise explanatory models of policy change, including that adapted to health policy experience (such as the Shiffman and Smith framework^12^), as well as that addressing particular phenomena, such as the array of relevant power theory.^13^ As Sheaff^4^ argues, a bricolage approach may be best in conducting such research, drawing from a range of theory, both from beyond and within the health policy terrain.

##  Second, is using theory the most important factor in deepening research about health policy processes? 

 We argue that it is not. Various reviews of the existing literature make clear that it is instead important to pay more attention to considering what study designs are best suited to researching complex policy processes that evolve over time, and to the selection of data collection approaches and analytic steps.^7^ Adequately describing study methods in published papers is also important, though constrained by word limits and differing journal expectations. We must also recognize our own positionality as researchers, and its impacts on our access to, and understanding of, the policy environment.^7^ Health policy process research conducted in an LMIC setting should, then, be led by researchers from that setting, or by those who are very familiar with it due to deep and continuous engagement. Wider research addressing LMICs, perhaps considering global influences on LMIC policy environments, should also preferably be undertaken with researchers from LMIC settings to ensure fuller understanding. It is, therefore, critical to extend and strengthen the pool of those involved in health policy analysis research within LMICs in order to strengthen this field.^14^

##  Third, it is necessary to reflect on why, as researchers, we do this work and how theory assists in working towards this goal. In other words, what is the relevance of generating understanding of health and other policy processes? 

 In his commentary, Cairney^5^ challenges us to think *why* we are using theory – and to consider the practical value theory has for those engaged in bringing about policy change. In other recent work he and his co-author have suggested that “…in their quest for better theories, policy scholars have often treated their peers as their sole audience, with little incentive to think about their usefulness to wider audiences or practical implications” (p. 3).^15^ These scholars then suggest that developing the field requires a dialogue between theory and practice, in which theory is used to offer guidance to policy actors and these actors themselves shape theory-based knowledge.

 From this perspective, we see value in the continued and wide application of the HPAT, notwithstanding that, as noted when first published, it is “a highly simplified model of an extremely complex set of relationships” (p. 355).^6^

 This simplicity means it is a helpful frame for assisting those from very different disciplinary traditions, such as medicine, epidemiology, or clinical science, in considering the political dimensions of health policy change. It also offers value to novice health policy analysts, as shown by its wide use in LMICs.^16^ The HPAT can guide their initial steps into this area of work^14^ and as they investigate policy processes more deeply, they can combine it with a wider range of relevant theory. Further, as we had hoped, the HPAT has been used in prospective policy analysis and by various groups of “policy practitioners.” These uses include supporting policy advocacy,^17^ as well as assisting public health managers to think through the political dimensions of their roles in implementing policy change.^18^ The spreading use of the HPAT is, engaging wider and wider circles of those working in the health sector to recognize the influence of policy actors, power and process in dynamic policy processes. It provides a common frame for understanding these dynamics that can, then, support dialogue among researchers and various groups of practitioners as well as deepen collective insights and action.

## Conclusions

 We have argued that although theory plays a role in developing deeper understanding of health policy processes in LMICs, the theory used must be relevant to the specific contexts of countries, and to health policy. We also argue that greater attention must be paid to study design and to researcher positionality. However, to support policy change, we need to bridge the worlds of research and policy. Simple but enduring policy models offer frames that can catalyse the collective understanding and action need to address critical social problems. Given the immense economic and social inequities shaping every facet of our lives today, including health and well-being, it is imperative to move beyond research to action. As one of us proposed over 20 years ago:

 “If we as health workers, or as teachers, or students, or civil servants, do not feel that we, and the groups or organisations which we belong to, have some power to alter the policy that affects our lives, or the lives of those around us, why get up in the morning?” (p. 10).^19^

## Ethical issues

 Not applicable.

## Competing interests

 Authors declare that they have no competing interests.
